# Case Report: Utility of brain [^18^F]FDG PET/CT in the diagnosis of Sydenham's chorea

**DOI:** 10.3389/fnume.2024.1527150

**Published:** 2024-12-24

**Authors:** Abel Dambrain, Charles Boursot, Kévin Cohen Tannugi, Julien Reichart, Franck Lacoeuille

**Affiliations:** ^1^ Department of Nuclear Medicine, University Hospital Center, Angers, France; ^2^ Department of Nuclear Medicine, Le Mans Hospital Center, Le Mans, France; ^3^ Radiopharmacist, CRCI2NA-Inserm UMR1307/CNRS UMR 6075, University of Angers, Angers, France

**Keywords:** PET, Sydenham's, brain, chorea, epilepsy

## Abstract

Sydenham's chorea is an autoimmune reaction against cerebral basal ganglia associated with rheumatic fever, caused by group A beta-hemolytic streptococcus infection. Diagnosis of this condition is difficult because of significant delay between infection onset and symptoms presentation, resulting in few positive biological tests or imaging exams. We report the case of a nine-year-old boy exhibiting hemicorporal abnormal movements with tics for whom [^18^F]FDG PET/CT exam allowed to make the diagnosis, associated with anti-DNase B elevation. Other biology, spinal tap, EEG and imaging modality like MRI or scanner, were non-contributory.

## Introduction

Sydenham's chorea is a late manifestation of Group A beta-hemolytic streptococci. This condition is rare in developed countries but remains prevalent in poorer regions (1,1,2).

The symptoms are alarming, with dysarthria, involuntary movements, and even paralysis (1–3). The diagnosis is difficult because requires several criteria. Streptococcal throat cultures and serological tests for recent streptococcal infection are often negative. CT scans are usually normal, but MRI is a good exam to exclude a stroke and help to diagnose Sydenham's chorea. All other tests, such as spinal tap or electroencephalogram, are usually normal (4, 5).

Antistreptolysin O (ASO) becomes positive 3–5 weeks after the initial pharyngitis, while anti-DNase B becomes positive 8–12 weeks later. Both remain detectable for several weeks to a few months, with anti-DNase B staying positive longer than ASO (2, 3, 6). However, obtaining immunological results can take several days, whereas rapid diagnostic guidance is desirable.

We report the case of a 9-year-old boy presenting with dysarthria and hemichorea, for whom all additional examinations, particularly biological tests and MRI, were non-contributory. While awaiting immunological results, a [^18^F]FDG PET scan was performed, providing arguments for Sydenham's chorea, which was confirmed a few days later by a positive anti-DNase B test. The contribution of the nuclear medicine exam to management patient was major, enabling the early introduction of corticosteroid therapy. Therefore, we emphasise the crucial role of [^18^F]FDG PET in the diagnosis of Sydenham's chorea when other modalities are negative.

## Case

A nine-year-old boy with no prior medical history or recent acute events was admitted to the hospital for right hemicorporal chorea, without any associated sensory or motor deficits. The child displayed uncoordinated movements, difficulty writing, a staggering gait, and some jaw and eye tics.

Initial urgent tests, including a CT scan and an MRI, showed no abnormalities, ruling out stroke. Further investigations, including EEG, basic blood tests (notably CRP and autoimmune markers), and cerebrospinal fluid analysis (with autoimmune tests and HSV PCR), also came back normal or non-specifics.

**Figure 1:**
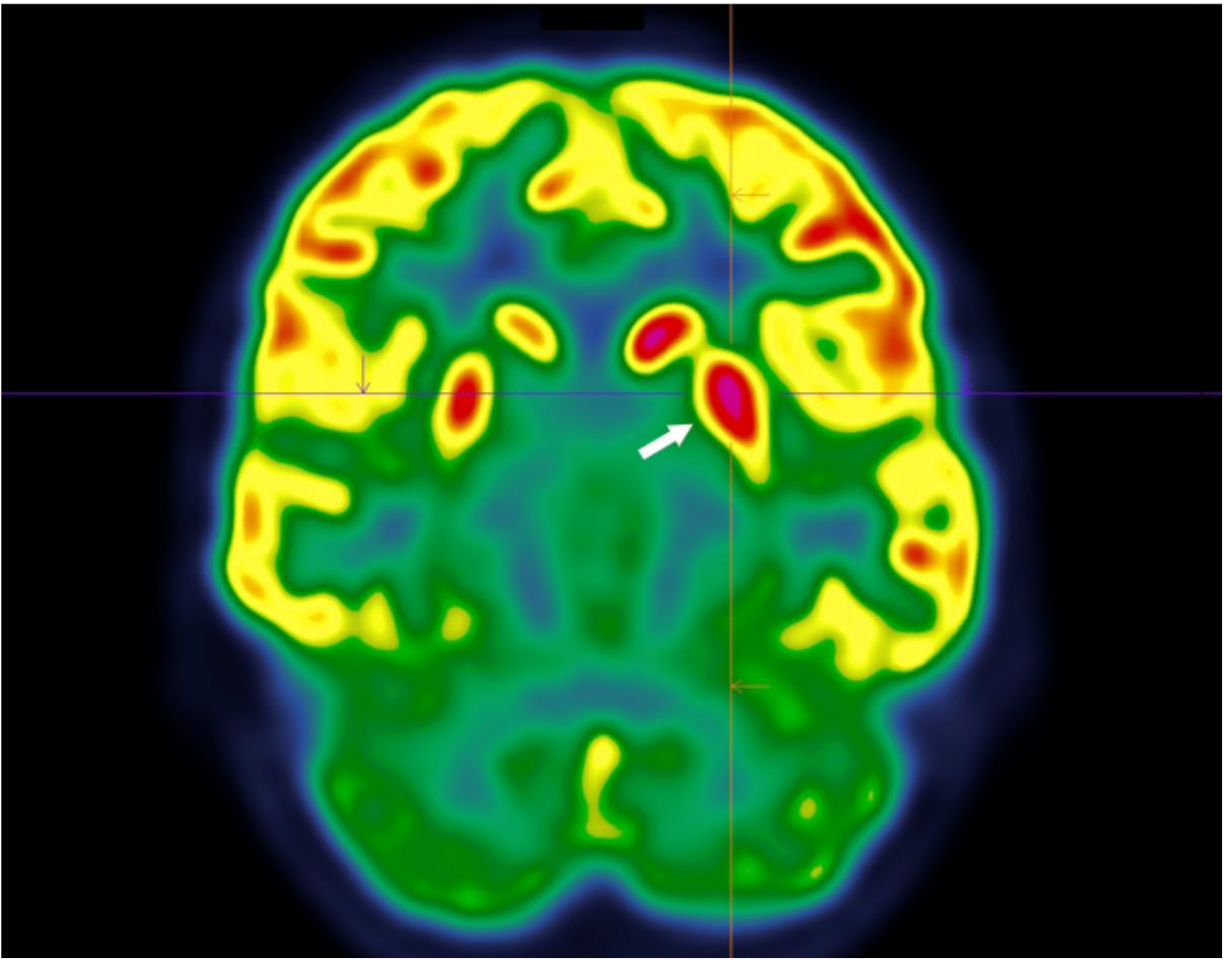
18F-FDG PET scan focused on the basal ganglia region showing asymmetry of the caudate nuclei and putamen, with predominant fixation on the left.

Despite normal test results, Sydenham's chorea was suspected by the paediatrician, leading to the performance of a cerebral [^18^F]FDG PET/CT eight days after symptom onset. A ten-minute focused brain acquisition revealed a moderate to intense asymmetrical hypermetabolism of the left caudate nucleus, extending into the ipsilateral putamen and pallidum (Figure 1, arrows), which was compatible with the diagnosis. Finally, ASO (antistreptolysin O) was normal, but anti-DNase B was positive and confirmed the diagnosis two weeks later.

Treatment with prednisolone was initiated, resulting in stabilization and a subsequent reduction in chorea. The patient, initially excited and unable to remain still, quickly calmed down after the treatment was started. All these elements confirmed the diagnosis of Sydenham's chorea.

## Discussion

Sydenham's chorea can develop following infection with Group A beta-hemolytic streptococci and is one of the main causes of chorea in children between 5 and 15 years old. Despite its continued prevalence in developing countries where access to amoxicillin is limited, Sydenham's chorea has significantly decreased in countries with better accessibility to antibiotics and healthcare (1, 2).

It appears in 10–30% of cases of rheumatic fever and predominantly affects females (approximately 70% of cases) (1, 7, 8). Clinical manifestations include choreiform motions, characterized by hyperkinetic movements typically affecting the face and limbs, which disappear during sleep. These manifestations are associated with psychiatric syndromes such as anxiety, depression, and obsessive-compulsive symptoms, as well as physical sensations of joints tension, tingling, and trembling sensations on the skin (7, 9, 10).

The pathophysiology is mainly explained by a cross-reactivity between antibodies against Group A *β*-hemolytic streptococcus and antigens of basal ganglia neurons. This autoimmune reaction leads to inflammation, resulting in the induction of kinase II enzymes and subsequent release of dopamine, resulting in abnormal motor movements (9, 10).

No single test provides a definitive diagnosis of Sydenham's chorea, which may appear several months after the initial infection (3). Thus, often the streptococcal throat culture and serology are negative. Diagnosis is typically guided by antistreptolysin O or anti-DNase B which remain elevated for weeks to months, but these tests are usually performed in a reference centre and results may takes a long time.

During the acute phase of the pathology, CT scans are usually normal, but MRI can reveal abnormalities in the basal ganglia, which appear enlarged with increased signal intensity on T2-weighted images (5). Persistence of signal abnormalities is associated with a greater number of recurrences (2, 4).

On [^18^F]FDG PET scans, an asymmetrical hypermetabolism of the striatum, affecting the caudate nucleus and the lentiform nucleus, can be detected, disappearing a few months later (11, 12).

Prevention of this condition is based on early penicillin treatment (1), and when Sydenham's chorea is diagnosed, high-dose corticosteroids, antiepileptics, and sometimes dopamine antagonists may be used (9). In severe cases, immunotherapy with immunoglobulins and plasmapheresis may be considered (13).

## Conclusion

Our case supports the importance of [^18^F]FDG PET in the early diagnosis of Sydenham's chorea when the disease is suspected and other exams are negative, with the aim of introducing corticosteroid therapy as soon as possible.

## Data Availability

The original contributions presented in the study are included in the article/Supplementary Material, further inquiries can be directed to the corresponding author.
